# Association of quantitative radiomic shape features with functional outcome after surgery for primary sporadic dorsal spinal meningiomas

**DOI:** 10.3389/fsurg.2023.1303128

**Published:** 2023-12-19

**Authors:** Martin Vychopen, Felix Arlt, Florian Wilhelmy, Clemens Seidel, Alonso Barrantes-Freer, Erdem Güresir, Johannes Wach

**Affiliations:** ^1^ Department of Neurosurgery, University of Leipzig Medical Center, Leipzig, Germany; ^2^ Cancer Center Central Germany, Partner Site Leipzig, Leipzig, Germany; ^3^ Department of Radiation Oncology, University of Leipzig Medical Center, Leipzig, Germany; ^4^ Paul Flechsig Institue of Neuropathology, University of Leipzig Medical Center, Leipzig, Germany

**Keywords:** functional outcome, shape, spinal meningioma, sphericity, surgery

## Abstract

**Objective:**

Spinal meningiomas (SM) account for 25%–46% of all primary spinal tumors and show an excellent long-term disease control in case of complete resection. Therefore, the postoperative functional outcome is of high importance. To date, reports on dorsally located SM are scarce. Moreover, the impact of radiomics shape features on the functional outcome after surgery for primary dorsal SMs has not been analyzed yet.

**Methods:**

We retrospectively performed an analysis of shape-based radiomic features in 3D slicer software and quantified the tumor volume, surface area, sphericity, surface area to volume ratio and tumor canal ratio. Subsequently, we evaluated the correlation between the radinomic parameters and the postoperative outcome according to Modified Japanese Orthopedic Association (mJOA) score.

**Results:**

Between 2010 and 2022, we identified 24 Females and 2 Males operated on dorsal SMs in our institutional database. The most common SM localization was thoracic spine (*n* = 20), followed by cervical (*n* = 4), and lumbar (*n* = 2). The univariate analysis and the receiver operating characteristic (ROC) analysis showed a strong diagnostic performance of sphericity in the prediction of postoperative functional outcome based on mJOA score (AUC of 0.79, sphericity cut-of value 0.738; *p *= 0.01). Subsequently, the patients were divided into two groups (mJOA improved vs. mJOA stable/worsened). Patients with improved mJOA score showed significantly higher sphericity (0.79 ± 0.1 vs. 0.70^ ^± 1.0; *p* = 0.03). Finally, we divided the cohort based on sphericity (<0.738 and ≥0.738). The group with higher sphericity exhibited a significantly higher positive mJOA difference 3 months postoperatively (16.6 ± 1.4 vs. 14.8 ± 3.7; *p* = 0.03).

**Conclusion:**

In our study investigating primary sporadic dorsal SMs, we demonstrated that a higher degree of sphericity may be a positive predictor of postoperative improvement, as indicated by the mJOA score.

## 1. Introduction

Spinal meningioma (SM) accounts for 25%–46% of all primary spinal tumors, while comprising 12% of all meningiomas (1–5). Notably, 90% of SMs are intradural, 5% are extradural, and the remaining 5% have both intra- and extradural components. SMs show a female predominance, and most cases are sporadic, despite known associations with neurofibromatosis type 2 or prior ionizing radiation exposure (6–8). SMs are further classified as either anterior/anterolateral to or posterior/posterolateral to the dentate ligament (9). In view of the reported high amount (95.7%) of World Health Organization (WHO grade) 1 meningiomas achieving excellent long-term tumor control after complete resection, preserving neurological function is of paramount importance (10).

SMs located anteriorly to the denticulated ligament are considered more challenging to resect and pose a risk factor for perioperative neurological deterioration (11, 12). In contrast, only a limited number of studies have focused exclusively on dorsally located SMs, despite a large-scale bicentric study reported comparable postoperative functional outcomes between ventrally and dorsally located SMS (8, 13). Recently, radiomics of the preoperative meningioma shape were observed to be significantly associated with the WHO grade of cranial meningiomas and with the postoperative cranial nerve functioning after surgery for medial sphenoid wing meningiomas (14, 15).

However, the impact of radiomics based shape features on postoperative neurological functioning in surgery for solely dorsally located SM has not been analyzed so far. The present retrospective investigation examines the role of shape features with regard to postoperative morbidity and neuropathological characteristics.

## 2. Methods

### 2.1. Study design and inclusion criteria

This study retrospectively reviews consecutive patients with primary sporadic SM who had undergone surgical resection between 2010 and 2022. The study was conducted according to the guidelines of the Declaration of Helsinki and approved by the Ethics Committee of the Medical Faculty of the University of Leipzig (No. 159/23-ek). Informed consent for scientific use of anonymized data was signed by all patients. Patients with dorsal SMs located below the foramen magnum were included. Patients with anterior foramen magnum meningiomas, craniocervical meningiomas with intracranial extension, ventrally located SMs, recurrent SM after radiotherapy, and patients with a neurofibromatosis type II were excluded due to the differences regarding clinical signs, histopathology, and therapy (3, 7, 12, 16, 17). Primary sporadic SMs localized posterior to the dentate ligament were considered as dorsal SM and included in this analysis (9).

### 2.2. Data Recording

To assess the patient characteristics, we analyzed the demographic data, laboratory tests on admission, comorbidities, preoperative neurological status according to modified Japanese Orthopedic Association (mJOA) score and retrospectively reviewed the patient charts to determine the modified McCormick Scale (mMCs). We also examined the operation protocols to evaluate the duration, blood loss, the use of the drains and the extent of the tumor resection. To define the extent of resection, we used the Simpson classification system in line with the European Association of Neuro-Oncology (EANO) (18). The histological reports were analyzed for WHO Grade, Molecular Immunology Borstel (MIB)-1 index and the number of mitotic figures. Finally, we reviewed the outpatient reports to assess the postoperative outcome.

### 2.3. Image analysis and data extraction

Image analysis of preoperative MRI scans using T1-weighted contrast enhanced and T2-weighted images was performed. We quantified the shape features of the lesion using the segment statistics including tumor volume, tumor surface area, sphericity, tumor surface area to volume ratio, and tumor canal ratio (formula: (tumor volume/spinal canal volume) × 100) (19). The Hakon-Wadell method was used to calculate sphericity (20). The following formula was used to determine the sphericity: Sphericity = π^1/3^(6 × tumor volume)^2/3^/surface area (20). The value of sphericity ranges from 0 to 1 with 1 indicating a perfectly spherical shape. The reviewer of the images was blinded to clinical data of the patients.

**Figure 1:**
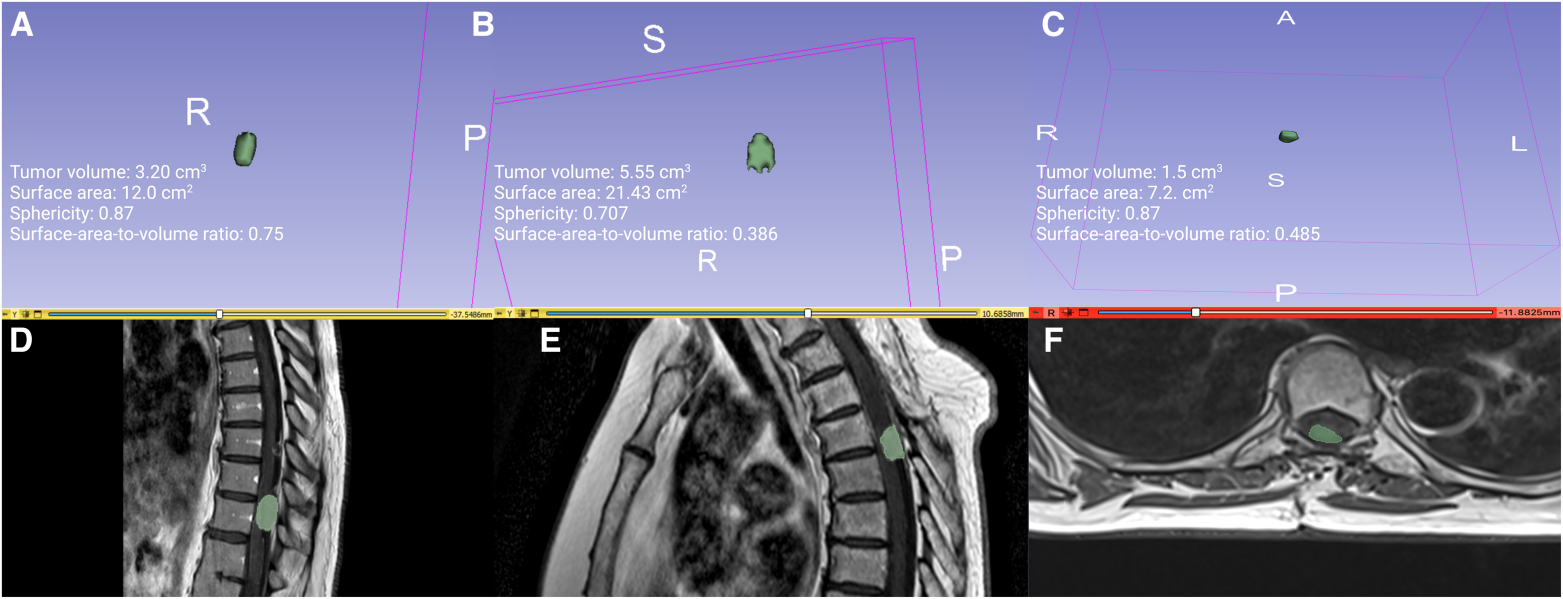
(**A,D**) illustrates a regularly shaped dorsal primary sporadic SM with the shape-based radiomic features tumor volume, surface area, sphericity, and surface-area-to-volume ratio, whereas (**B,E**) show an irregularly shaped dorsally located primary sporadic SM. Illustratory case**s** of tumor segmentation with 3D-Slicer (version 5.2.1, Surgical Planning Laboratory, Harvard University, US). (**C,F**) show a regularly shaped dorsal primary sporadic SM with the 3D volume and the corresponding axial T1-gadolinium enhanced sequence.

The medical image analysis and visualization was performed using 3D slicer software (version 5.2.1, Surgical Planning Laboratory, Harvard University, USA) (21). The segmentation of the lesion was performed in a semi-automatic fashion using the “Fast Marching” method according to Pichon et al. (22). For detailed information, see Figure 1.

### 2.4. Surgical procedure

Indications for surgery included pain, neurological dysfunctions, and spinal cord compression. Surgical management was dependent on the site of dural attachment of the tumor, tumor size, and the affected segments. The usual workflow involved laminectomy, dural opening and en-bloc or piecemeal resection of the tumor. After removing the tumor, the dural attachement was coagulated using bipolar coagulation forceps and dura suture was supported with TachoSil(®) (Takeda Austria GmbH, Linz, Austria).

### 2.5. Immunohistochemistry

Paraffin sections were stained with hematoxylin and eosin (H&E). Immunohistochemical investigation was performed on the specimen using the Molecular Immunology Borstel-I (MIB-I, DAKO, Glostrup, Denmark) antibody to detect the Ki67 antigen and calculate the MIB-I index by the average method as previously described (30).

### 2.6. Statistics

Data were organized and analyzed using SPSS for Windows (version 29.0; IBM Corp., Armonk, NY, USA). Kolmogorov-Smirnov (KS) test were performed to investigate distributions. Normally distributed data are reported as the mean with the standard deviation (SD). Receiver-operating characteristic (ROC) curves were constructed to analyze the diagnostic value of the quantitative shape-based feature sphericity in association with the postoperative course of the mJOA following surgery for dorsal SM (23). Cut-off values for sphericity were determined using the ROC analysis. Preoperative demographics, tumor volume, tumor surface area, sphericity, surface-to-volume ratio, histopathological, and immunohistochemical features were compared between patients with worsened and stable or improved mJOA at 3-months after surgery using Fisher's exact test (two-sided) for categorical values and independent *t*-test for continuous values. Violin-Plots displaying sphericity among those SM patients with worsened and stable or improved mJOA at 3-months after surgery were created using Prism 8 for macOS (Version 8.4.3, GraphPad Software, San Diego, CA, USA).

## 3. Results

### 3.1. Patient characteristics

Patient characteristic showed an unequal distribution of sex with 24 Females and 2 Males. The median age was 67.5 (IQR 57.3–80.0). The mJOA score preoperatively amounted to 15.0 (IQR 13.0–17.0) and the median KPS was 80 (IQR 70–90). The median preoperative mMCs was 2.0 (IQR: 1.0–4.0).

Most of the meningiomas were localized in thoracic part of the spine (*n* = 20), followed by cervical (*n* = 4) and lumbar (*n* = 2). The median tumor volume was 2.9 cm^3^ (IQR 1.4–3.9), with median surface area of 11.7 cm^2^ (8.5–16.1). The surface to volume ratio was 0.47 (IQR 0.40–0.56), and sphericity was 0.71 (IQR 0.66–0.80). Two SMs showed signs of calcification, and only one SM was cystic.

Postoperative histological examination revealed 21 WHO Grade 1 SMs and 5 WHO Grade 2 SMs. The median MIB-1 index was 4.25 (IQR 3.0–5.25) and the median number of mitotic figures was 3 (IQR 2–8.75).

According to operation protocols, Simpson Grade I resection was achieved in 1 case, Simpson Grade II resection in 23 cases and Simpson Grade IV resection in 2 cases. For detailed information, please refer to Table 1.

**Table 1:** Patient characteristics (*n* = 26).

Median age (IQR) (in year)	67.5 (57.3–80.0)
Sex	
Female	24 (92.3%)
Male	2 (7.7%)
Median preoperative mJOA (IQR)	15.0 (13.0–17.0)
Median preoperative mMCs (IQR)	2.0 (1.0–4.0)
Median preoperative KPS (IQR)	80 (70–90)
Spinal level	
Cervical	4 (15.4%)
Thoracic	20 (76.9%)
Lumbar	2 (7.7%)
Tumor volume, cm^3^, median (IQR)	2.9 cm^3^ (1.4–3.9)
Tumor surface area, cm^2^, median (IQR)	11.7 (8.5–16.1)
Tumor surface area to tumor volume ratio, median (IQR)	0.47 (0.40–0.56)
Tumor canal ratio, median (IQR)	65.3% (46.5%–76.5%)
Sphericity, median (IQR)	0.71 (0.66–0.80)
Calcification	2 (7.7%)
Cyst	1 (3.8%)
Simpson grade	
Simpson grade I	1 (3.8%)
Simpson grade II	23 (88.5%)
Simpson grade III	0 (0.0%)
Simpson grade IV	2 (7.7%)
WHO grade	
WHO grade 1	21 (80.8%)
WHO grade 2	5 (19.2%)
MIB-1 index, Median (IQR)	4.25 (3.0–5.25)
Mitotic figures, Median (IQR)	3.0 (2.0–8.75)

KPS, karnofsky performance status; MIB-1, molecular immunology borstel-1; SD, standard deviation; mJOA, modified Japanese orthopaedic association score; WHO, world health organization.

### 3.2. Sphericity in the prediction of neurological course

**Figure 2:**
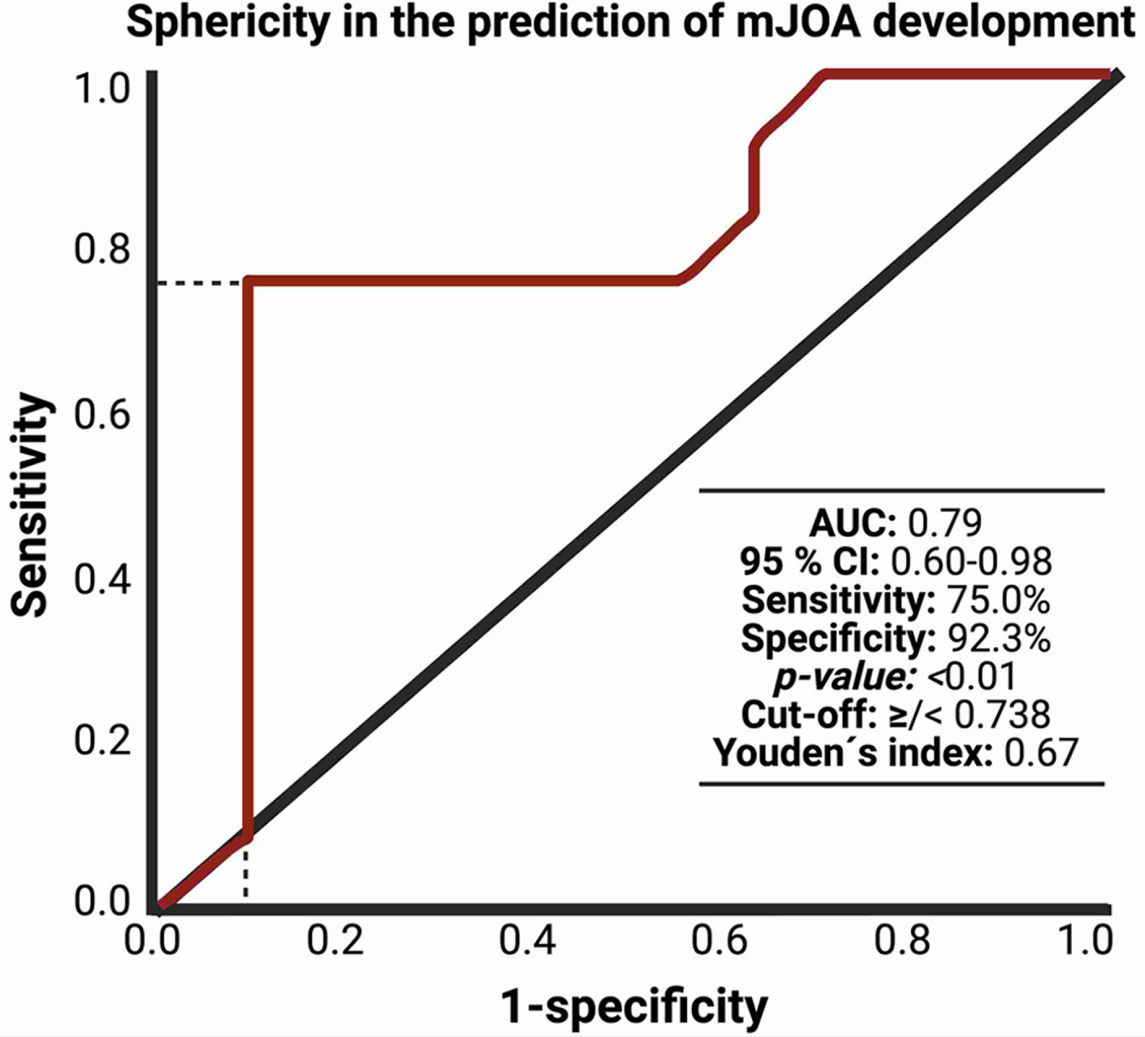
Receiver-operating characteristic curve (ROC) illustrating the ability of sphericity in the association with mJOA development within 3 months after surgery for dorsal primary sporadic spinal meningioma.

After performing the radiomic analysis with 3-D Slicer (Version 4.10), we conducted a ROC analysis of sphericity and mJOA difference (preoperative compared to 3 months postoperative). The analysis revealed an area under curve (AUC) of 0.79 with 75% sensitivity and 92.3% specificity and sphericity cut-off value of 0.738 (*p* = 0.01). For details on ROC-Curve analysis, refer to Figure 2.

**Figure 3:**
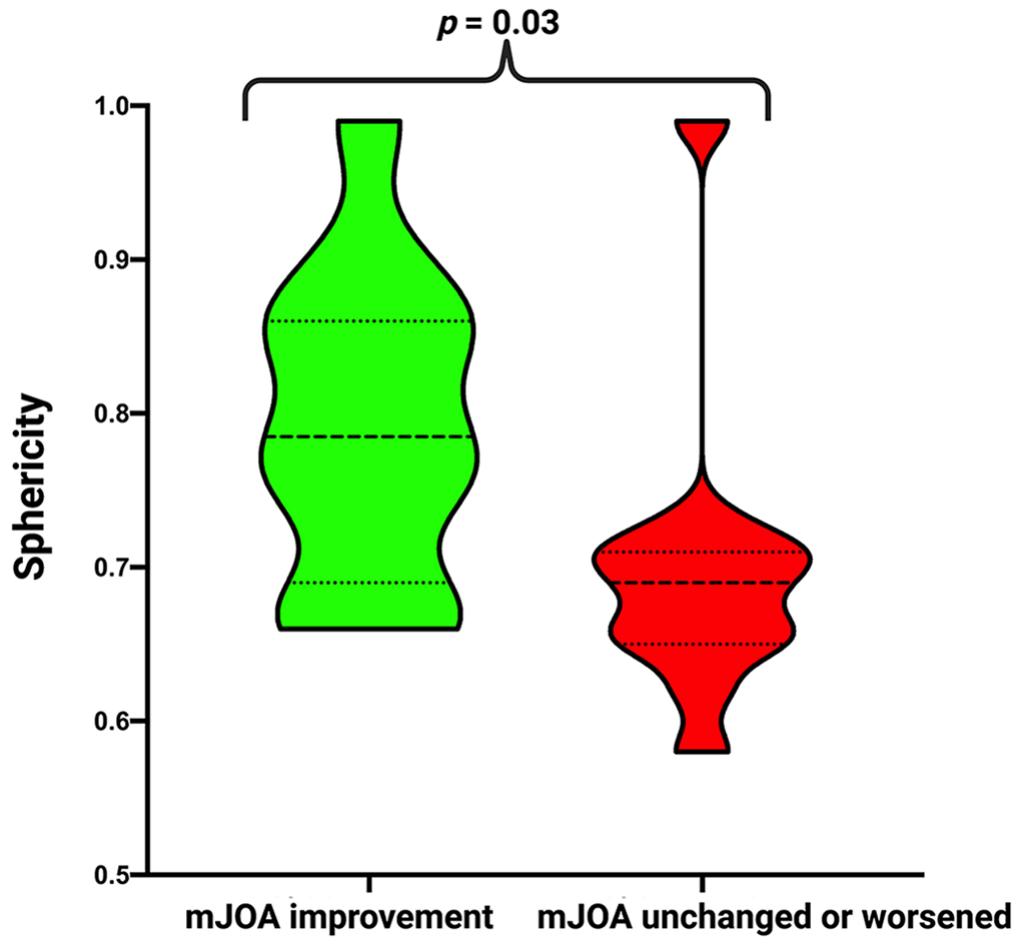
Truncated violin-plots displaying sphericity in patients with mJOA improvement (green) and with stable/worsened mJOA (red). Median values are presented by the thick black lines (*p*-values of the Student's *t*-test).

Subsequently, we divided the cohort into two groups according to postoperative mJOA score. We compared patients who showed mJOA improvement (delta: ≥1) to patients with stable or worse postoperative mJOA score (delta: <1). There was a significantly higher sphericity in patients in the improvement group (0.79 ± 0.1 vs. 0.70^ ^± 1.0; *p* = 0.03). We found no difference in age, sex, preoperative KPS and mJOA, tumor size, tumor area, surface to volume ratio, Simpson Grade, WHO grade, MIB-1 index and Mitotic figure count. For detailed information, refer to Table 2 and Figure 3.

**Table 2:** Comparison of patient characteristics in groups with improvement of mJOA or worsening/stable mJOA after 3 months compared with baseline mJOA after surgery for dorsal primary sporadic spinal meningioma [using Fisher's exact test (two-sided) and independent *t*-test].

Characteristics	mJOA improvement (delta: ≥1) (12/26; 48.0%)	mJOA stable or worsened (delta: <1) (13/25; 52.0%)	*p*-value
Age (years), mean ± SD	66.6 ± 15.6	65.8 ± 15.5	0.90
Sex			0.99
Female	11 (91.7%)	12 (92.3%)	
Male	1 (8.3%)	1 (7.7%)	
Preoperative KPS, mean ± SD	79.2 ± 13.1	77.7 ± 20.5	0.83
Preoperative mJOA, mean ± SD	14.7 ± 2.3	14.6 ± 3.5	0.97
Preoperative mMCs, mean ± SD	2.17 ± 1.3	2.46 ± 1.5	0.60
Sphericity, mean ± SD	0.79 ± 0.1	0.70^ ^± 1.0	*0*.*03*
Tumor volume, cm^3^, mean ± SD	2.5 ± 1.3	3.0 ± 1.9	0.46
Tumor area, mm^2,^ mean ± SD	10.9 ± 4.1	13.9 ± 7.1	0.21
Surface-to-volume ratio, mean ± SD	0.49 ± 0.16	0.55 ± 0.19	0.42
Tumor canal ratio, mean ± SD	64.3 ± 18.2	58.3 ± 28.0	0.54
Simpson grade			0.68
≤III	12 (100.0%)	11 (84.6%)	
>III	0 (0.0%)	2 (15.4%)	
WHO grade			0.48
1	10 (83.3%)	10 (76.9%)	
2	2 (16.7%)	3 (23.1%)	
MIB-1 index, mean ± SD	4.8 ± 2.3	5.6 ± 4.9	0.60
Mitotic figures, mean ± SD	3.0 ± 1.6	7.0 ± 6.4	0.16

KPS, karnofsky performance status; MIB-1, molecular immunology borstel-1; SD, standard deviation; mJOA, modified Japanese orthopaedic association score; WHO, world health organization.

*P*-values in italic represent statistically significant results.

Finally, we divided the cohort into two groups according to postoperative mJOA score. We compared patients who showed mJOA improvement (delta: ≥ 1) to patients with stable or worse postoperative mJOA score (delta: <1). There was a significantly higher sphericity in those patients, who showed an improvement of the functional outcome at 3-months after surgery (0.79 ± 0.1 vs. 0.70^ ^± 1.0; *p* = 0.03). Patients with a low sphericity also had a significantly higher mMCs at 3 months (2.47 +/−1.56 vs. 1.50 +/−0.71, *p* = 0.047) and a poorer outcome if the intraindividual delta (mMCs 3 months vs. mMCs preoperative: 0.13 +/− 1.12 vs. −0.80 +/− 0.79, *p* = 0.03) was assessed. The baseline functioning displayed by the preoperative mJOA or mMCs did not differ between those with low or high sphericity. We found no differences in age, sex, preoperative KPS and mJOA, tumor size, tumor area, surface to volume ratio, Simpson Grade, WHO grade, MIB-1 index and mitotic figure count among those with decreased or normal sphericity. For detailed information, refer to Table 3 and Figure 3.

**Table 3:** Baseline clinical, pathologic, and imaging characteristics in dorsal spinal meningioma patients with a decreased and normal sphericity [using Fisher's exact test (two-sided) and independent *t*-test].

Characteristics	Sphericity < 0.738% (16/26; 61.5%)	Sphericity ≥ 0.738 (10/26; 38.5%)	*p*-value
Age (years), mean ± SD	64.2 ± 16.4	70.2 ± 12.4	0.33
Sex			0.20
Female	14 (87.5%)	10 (100.0%)	
Male	2 (12.5%)	0 (0.0%)	
Preoperative mJOA, mean ± SD	14.5 ± 3.2	14.8 ± 2.6	0.80
Preoperative mMCs, mean ± SD	2.31 ± 1.4	2.30 ± 1.3	0.98
Preoperative KPS, mean ± SD	78.0 ± 18.46	79.0 ± 15.2	0.89
Simpson grade			0.99
≤III	15 (93.75%)	9 (90.0%)	
>III	1 (6.25%)	1 (10.0%)	
WHO grade			0.99
1	13 (81.25%)	8 (80.0%)	
2	3 (18.75%)	2 (20.0%)	
MIB-1 index, mean ± SD	4.8 ± 4.6	5.5 ± 1.9	0.68
Mitotic figures, mean ± SD	5.9 ± 6.5	4.6 ± 3.4	0.70
Length of stay, days, mean ± SD	9.4 ± 5.6	9.7 ± 4.3	0.89
mJOA at 3-months, mean ± SD	14.8 ± 3.7	16.6 ± 1.4	0.10
Delta of mJOA at 3-months compared with baseline, mean ± SD	0.3 ± 1.8	1.8 ± 1.4	*0*.*03*
mMCs at 3-months, mean ± SD	2.47 ± 1.56	1.50 ± 0.71	*0*.*047*
Delta of mMCs at 3-months compared with baseline, mean ± SD	0.13 ± 1.12	−0.80 ± 0.79	*0*.*03*

KPS, karnofsky performance status; MIB-1, molecular immunology borstel-1; SD, standard deviation; mJOA, modified Japanese orthopaedic association score; WHO, world health organization.

The italic values represent statistically significant values.

## 4. Discussion

The present investigation analyzed shaped radiomic features based on shape to determine their association with postoperative neurological outcome and neuropathological characteristics in primary sporadic dorsal SMs. Our findings revealed that reduce sphericity, indicative of a more irregular SM shape, is significantly associated with worsened postoperative neurological outcome. However, we did not observe any significant association between shape and neuropathological factors.

Generally, resection of primary sporadic dorsal SM is considered a safe procedure, and the majority of patients experience significant improvement in their neurological outcomes. According to a recent systematic review, 65.2% of SM patients showed improvement, while 28.8% remained unchanged in terms of neurological functioning (10). However, the literature reports a range from 0% to 15.2% of patients experiencing postoperative worsening of their neurological functions (10). Poor postoperative neurological functioning is of paramount importance concerning health-related quality of life and cost-effectiveness, as individuals with worse neurological functioning might require prolonged hospital treatment, postoperative rehabilitation programs, and experience delayed return to work (24). Several factors have been described as predictive of the postoperative neurological status of SM patients. For instance, basic demographic feature such as older age, preoperative degree and duration of symptoms have been identified as risk factors for an unfavorable neurological outcome (24–28). However, in the present anatomic subgroup of dorsal primary sporadic SM, no heterogeneous distribution of age, preoperative functioning and symptom duration has been observed among SM patients with improved, worsened, or stable neurological functioning. These predictors have been described in different SM cohorts, and there have been various time endpoints used for determining postoperative functioning. In a retrospective single institutional study by Viereck et al. (29), patients with intradural extramedullary spinal tumors were followed-up for more than 12 months using the generic EQ-5D questionnaire. The study found that mobility and self-care worsened within 1 month following surgery, but significantly improved between the 3-to 12-month follow-up period. Therefore, using the 3-month time endpoint for evaluating the course of the mJOA score in the present study appears to be reliable. We found that patients with a low sphericity reflecting those patients with an irregularly shaped SM had a poorer neurological recovery at 3-months according to both mJOA score and mMCs. Furthermore, we found that the baseline neurological functioning did not differ between those a worsened, stable, or improved neurological function at 3-months after surgical resection. The preoperative neurological functioning has been described as an important predictor of outcome (10, 25). However, most of these studies do not exclusively focus on the dorsally located SMs.

The present manuscript represents the first analysis of the shape-based radiomic feature, sphericity, investigating its correlation with neurological functioning. It was observed that irregularly shaped dorsal primary sporadic SMs were significantly associated with worsened functional outcomes at 3-months after surgery. Meningiomas are often categorized into regularly or irregularly shaped tumors based on the characteristics of their margins and infiltrative growth patterns. The irregular shape is assumed to be caused by significant variations in the rate of cell proliferation within different subregions of the tumor, with certain areas displaying notably accelerated growth rates (30, 31). Hence, the shape of meningiomas has been proposed as a potential indicator of meningioma grade (32). Sphericity, a measurable shape characteristic derived from magnetic resonance images, has been observed to correlate with survival outcomes in glioblastoma (33). In an analysis involving 303 patients who underwent surgical removal of non-skull or skull base meningiomas classified as WHO grades 1–3, it was revealed that decreased sphericity significantly predicted both local meningioma regrowth and survival. In a previous institutional series, we could also identify sphericity as a surrogate parameter regarding postoperative cranial nerve functioning after surgery for medial sphenoid wing meningioma (15). Furthermore, an irregular shape was found to be significantly associated with increased proliferative activity, as reflected by the MIB-1 index, in medial sphenoid wing meningiomas (15). However, in the present series, we could not observe a correlation between the shape of primary dorsal SMs and the number of mitotic figures or the MIB-1 index. The strong correlation between meningioma shape and WHO grading of 126 cranial tumors has been found by Popadic et al. (14). However, this investigation exclusively focused on cranial meningiomas. Therefore, the association between shape and WHO grading in spinal meningiomas has not been confirmed yet. To further investigate and address the correlation between spinal meningioma shape and molecular characteristics driving proliferation large-scale cohorts are needed. Moreover, the impact of quantitative shape measures, such as sphericity, on progression-free survival in SMs, needs to be investigated in a large-scale cohort.

We exclusively investigated this shape-based quantitative feature regarding the outcome in dorsally located primary sporadic SM because ventral dural attachment is frequently analyzed and suggested as a potential risk factor for worsened outcome (1, 11, 12). Furthermore, in dorsally located primary sporadic SMs, division of the dentate ligament and lateralization of the myelon are not necessary. These crucial steps in the surgical approach of the resection of ventrally located SMs might be essential confounders in a small cohort investigating shape-based radiomics in primary sporadic SMs regarding functional outcome (34). Hence, shape-based radiomic features seem to be an important factor when informing patients and their relatives about the risks and potential outcomes after surgery for dorsally located primary sporadic SM. Future large-scale studies will need to externally validate our findings. Additionally, further studies should evaluate whether shape-related features are also significant in relation to progression-free survival and postoperative follow-up imaging.

### 4.1. Limitations

The main limitation of the present study is the retrospective nature and single-center design, with limited sample size. However, the present study considered highly selective inclusion criteria in order to focus the investigation on a precise anatomical subgroup of SMs. Further large-scale studies will have to evaluate our findings using multivariable analyses and investigate whether those quantitative shape features can be correlated with intraoperative findings such as the attachments to arachnoid webs or invasive growth of SMs.

## 5. Conclusion

The present study shows the correlation of sphericity with the postoperative outcome of the patients with dorsally attached primary sporadic spinal meningioma. This shape-based radiomic parameter could potentially serve as a valuable supporting parameter in indication of the operative resection and preoperative risk-evaluation.

## Data Availability

The original contributions presented in the study are included in the article/Supplementary Material, further inquiries can be directed to the corresponding author.
