# Supplementary figures and images for: Association of quantitative radiomic shape features with functional outcome after surgery for primary sporadic dorsal spinal meningiomas
Source: Front Surg. 2023 Dec 19;10:1303128. doi: 10.3389/fsurg.2023.1303128 (PMC10795533; doi:10.3389/fsurg.2023.1303128)

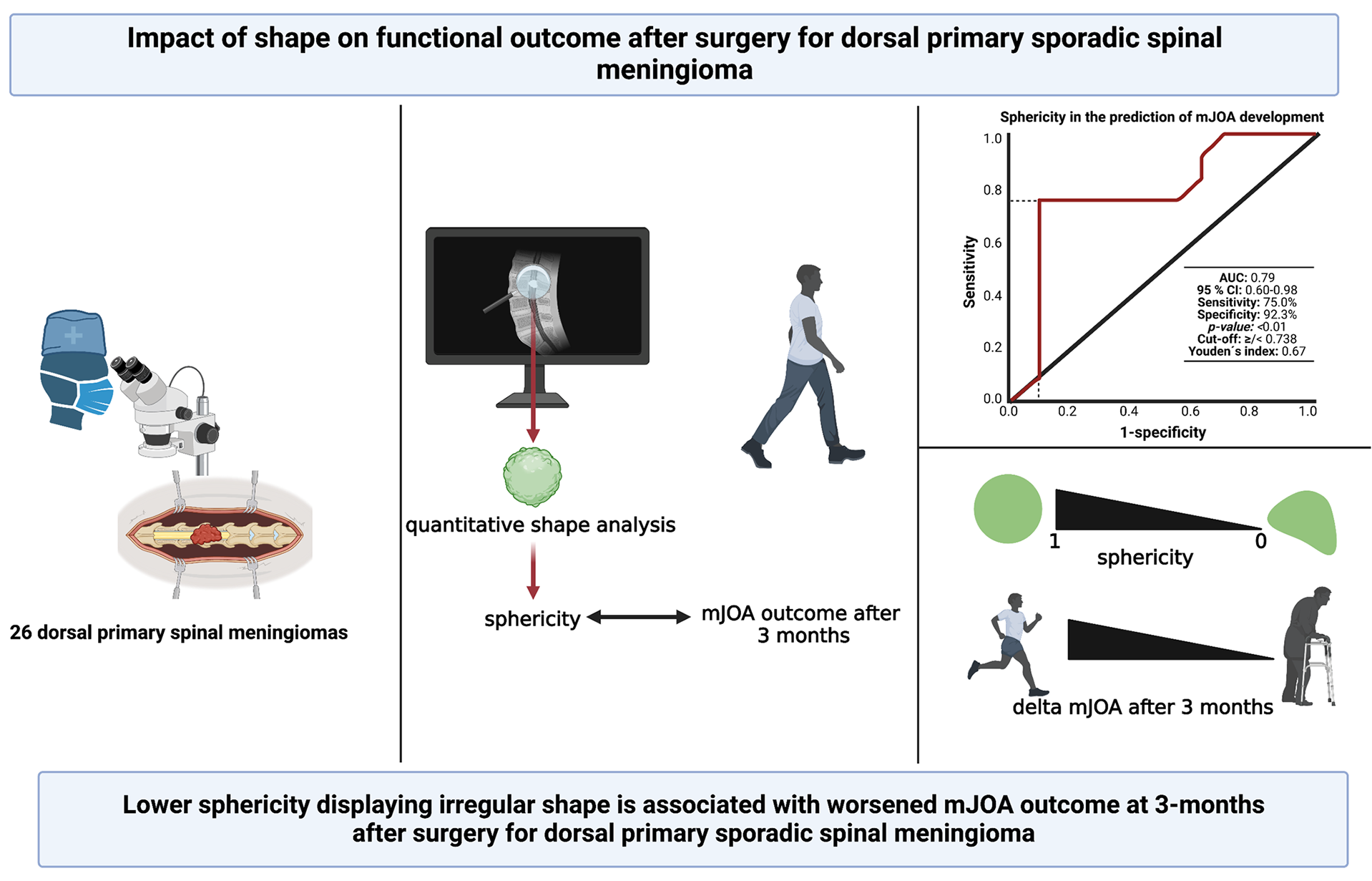

Supplement: Supplementary file 1 [file Image1.tif]
